# A Simple PB/LIE Free Energy Function Accurately Predicts the Peptide Binding Specificity of the Tiam1 PDZ Domain

**DOI:** 10.3389/fmolb.2017.00065

**Published:** 2017-09-26

**Authors:** Nicolas Panel, Young Joo Sun, Ernesto J. Fuentes, Thomas Simonson

**Affiliations:** ^1^Laboratoire de Biochimie (CNRS UMR7654), Ecole Polytechnique, Palaiseau, France; ^2^Department of Biochemistry, Roy J. and Lucille A. Carver College of Medicine, University of Iowa, Iowa City, IA, United States; ^3^Holden Comprehensive Cancer Center, Iowa City, IA, United States

**Keywords:** molecular dynamics, continuum electrostatics, NAMD SOFTWARE, fluorescence anisotropy, peptide design

## Abstract

PDZ domains generally bind short amino acid sequences at the C-terminus of target proteins, and short peptides can be used as inhibitors or model ligands. Here, we used experimental binding assays and molecular dynamics simulations to characterize 51 complexes involving the Tiam1 PDZ domain and to test the performance of a semi-empirical free energy function. The free energy function combined a Poisson-Boltzmann (PB) continuum electrostatic term, a van der Waals interaction energy, and a surface area term. Each term was empirically weighted, giving a Linear Interaction Energy or “PB/LIE” free energy. The model yielded a mean unsigned deviation of 0.43 kcal/mol and a Pearson correlation of 0.64 between experimental and computed free energies, which was superior to a Null model that assumes all complexes have the same affinity. Analyses of the models support several experimental observations that indicate the orientation of the α_2_ helix is a critical determinant for peptide specificity. The models were also used to predict binding free energies for nine new variants, corresponding to point mutants of the Syndecan1 and Caspr4 peptides. The predictions did not reveal improved binding; however, they suggest that an unnatural amino acid could be used to increase protease resistance and peptide lifetimes *in vivo*. The overall performance of the model should allow its use in the design of new PDZ ligands in the future.

## 1. Introduction

Protein-protein interactions (PPIs) are important for the information flow within and between cells. A large and important class of PPIs are mediated by PDZ domains (for “post-synaptic density-95/discs large/zonula occludens-1”). These are small, ~90 amino acid domains, found in most organisms from bacteria to vertebrates. The human genome encodes over 250 PDZ-containing proteins, which participate in many cellular activities, including the control of cell migration, invasion, proliferation, and polarity. PDZ domains usually recognize a peptide segment at the target protein's C-terminus, which binds as a β-sheet extension (Shepherd and Fuentes, 2011; Subbaiah et al., 2011). Individual pockets within the domain are used to accommodate the side chains of peptide residues and provide binding specificity. PDZ domains can also recognize the peptide segments in isolation, and the PDZ-peptide complexes represent simpler model constructs to investigate binding. PDZ domains have been widely studied, with over 200 X-ray structures in the Protein Data Bank (PDB). However, our current understanding and ability to engineer PDZ-peptide interactions is still limited. Indeed, the binding affinity and specificity depend on many factors, including short-range interactions between the two partners, longer-range electrostatic interactions, dielectric shielding by protein and solvent, ordered waters in the binding site, the structure and flexibility of the unbound peptide, and the conformational dynamics of the PDZ domain. All these effects are difficult to quantify using experiments. Computer simulations are a complementary tool, well-suited to study solvated proteins and their structure, dynamics, and ligand binding. An important advantage of simulations is the possibility of decomposing overall binding free energies into contributions from microscopic interactions to determine their structural origin.

Here, we used molecular dynamics (MD) simulations to investigate the Tiam1 PDZ domain and its binding to a collection of peptides. Tiam1, or “T-cell lymphoma invasion and metastasis-1,” is a guanine nucleotide exchange factor that specifically activates the Rho-family GTPase Rac1 (Mertens et al., 2003). Tiam1 is overexpressed in several kinds of tumors (Xu et al., 2010; Li et al., 2016). The Tiam1 PDZ domain is a Class II domain, capable of binding synthetic peptides with an -X-Φ-X-Φ motif at their C-terminus, where Φ represents a hydrophobic amino acid. Our ligand collection contains 27 peptides, corresponding to the C-terminus of Syndecan, Caspr4, CADM1 and Neurexin proteins, several of their mutants, peptides from a combinatorial library (Shepherd et al., 2011), and a “library-consensus” peptide (Songyang et al., 1997). The complexes studied involve wildtype and seven Tiam1 mutants. X-ray structures are known for four of the complexes and the wildtype apo-protein. Experimental binding affinities are known for most of the complexes; for some others, binding is very weak and only a lower bound for the dissociation constant is known. While the available data have already given considerable insights, several effects are difficult to quantify with experiments alone. In particular, we hypothesize that subtle structural rearrangements in the solutes and solvent can account for the observed range of binding affinities, and that free energy simulations can provide structural insight. To test the latter hypothesis, we have examined the ability of the simulations to reproduce the known binding affinities. Specifically, we performed molecular dynamics (MD) simulations of 51 solvated complexes, then characterized them using a semi-empirical free energy function that contains a continuum electrostatic term, a solvent-accessible surface area term (SA), and a van der Waals interaction term. The electrostatic term used either a Poisson-Boltzmann (PB) or a Generalized Born (GB) representation of the system. We used the simulations to characterize the structure and dynamics of several complexes and to predict the binding affinities of some new variants. Conversely, the experimental dataset provide a challenging test for the free energy function and reveal some of its limitations.

The “PBSA/GBSA” class of free energy models, used here, has been widely studied, and many variants exist (Srinivasan et al., 1998; Brandsdal et al., 2003; Jorgensen, 2003; Carlsson et al., 2006; Simonson, 2007, 2013; Gallicchio and Levy, 2011; Baron and McCammon, 2013; Harris et al., 2015; Chakavorty et al., 2016; Wang et al., 2016; Katkova et al., 2017). The model has several adjustable parameters. Our variant uses three weights, one for each of the free energy terms. Very similar, “Linear Interaction Energy” (LIE) variants have been widely-used for protein-ligand affinities (Zhou et al., 2001; Brandsdal et al., 2003; Jorgensen, 2003; Carlsson et al., 2006). We refer to the present variants as PB/LIE and GB/LIE models, respectively. A difficulty with semi-empirical models is the need to parameterize with a sufficiently broad and representative set of experimental data. Here, we rely on a large number of known binding affinities (44 in all), which span a modest free energy range of about 2.2 kcal/mol (dissociation constants from about 10 to 450 μM). Some of the affinities were measured in this work, and one (involving a non-natural amino acid) was estimated from a non-empirical, alchemical free energy simulation approach, which does not involve any adjustable parameters (Simonson et al., 2002; Chipot et al., 2007). A broader, combinatorial library of several hundred peptides that bind the Tiam1 PDZ domain has also been characterized (Shepherd et al., 2011), indicating amino acid preferences at each position within the peptide ligand, although the precise affinities were not measured.

The ability of the semi-empirical models to reproduce the experimental trends and affinities represents a good test of the PBSA and GBSA class of models, and should be of general interest. The PB/LIE and GB/LIE models gave very similar results. The best PB/LIE model gave a mean unsigned error of 0.43 kcal/mol and a Pearson correlation of 0.64 between the computed and experimental values. A simple Null model gave the same mean error but no correlation. 99.94% out of 100,000 random, or “Scrambled” models also gave poorer results. The Scrambled models were obtained by associating each experimental binding affinity with an arbitrary complex within our dataset—*not* the one for which it was measured—then adjusting the free energy weights to minimize the rms deviation between the computed data and the scrambled experimental data. The model and MD simulations also provided structural information regarding the role of the α_2_ helix in specificity. A few of the MD structures were validated by running rigorous, alchemical free energy simulations: since these gave excellent agreement with experiment, we conclude that the sampled structures are correct. The simulations were used to predict the binding affinities of nine new variants, including eight point mutants of the natural peptide Syndecan1 (Sdc1) binding to the WT Tiam1 PDZ domain. Although none of the variants have increased binding compared to the WT:Sdc1 complex, we predict that an unnatural amino acid can be introduced at the C-terminus of both the Sdc1 and Caspr4 peptides without loss of binding. Such an amino acid might provide protease resistance and increase the peptide stability *in vivo*.

## 2. Methods

### 2.1. The semi-empirical free energy function

To obtain the binding free energy estimate Δ*G*, we used the following ansatz for the free energy, or “scoring” function:

(1) $$\Delta G=\alpha \Delta E_{\text{vdW}}+\beta \Delta G_{\text{elec}}+\gamma \Delta A+\delta$$

Here, α, β, and γ represent adjustable constants. Δ*G*_elec_ is an electrostatic free energy difference between the bound and unbound states, computed with either a PB or a GB model, and averaged over structural snapshots taken at regular intervals along an MD trajectory of the (explicitly) solvated complex. Δ*A* is the change in the solute molecular surface upon binding (which is negative), averaged over the MD snapshots. Δ*E*_vdW_ is the average van der Waals interaction energy between the protein and the peptide. Solute-solvent and solvent-solvent van der Waals contributions are not explicitly included. In all three free energy terms, to represent the unbound state, we took the snapshots from the MD trajectory of the *complex* and simply moved the protein and the peptide apart. The energies of the separated protein and peptide were then computed. This is referred to as the “single trajectory” approach. The last term, δ, is a constant that vanishes when we consider the *relative* binding free energies ΔΔ*G* of the various complexes, using the Tiam1:Sdc1 complex as reference. The MD trajectories were 40–100 ns long, depending on the rate of convergence of Δ*G*.

### 2.2. Structural models

We modeled 51 PDZ:peptide complexes based on four X-ray structures involving four peptides: Sdc1, Caspr4, Neurexin, and a “consensus” peptide that represents a typical sequence from a combinatorial peptide library (Songyang et al., 1997). The peptides were bound to either the wildtype Tiam1 PDZ domain (WT) or a variant containing four amino acid changes (quadruple mutant or QM). The four complexes are WT:Sdc1 (PDB 4GVD) (Liu et al., 2013), WT:consensus (PDB 3KZE) (Shepherd et al., 2010), QM:Caspr4 (PDB 4NXQ) (Liu et al., 2016), and QM:Neurexin (PDB 4NXR) (Liu et al., 2016). Ten other complexes involving Caspr4 or its F0A mutant were modeled starting from QM:Caspr4. Two complexes involving Neurexin were modeled starting from QM:Neurexin. All the other complexes were modeled starting from WT:Sdc1. In each case, mutated peptide or protein side chains were positioned using the Scwrl4 program (Krivov et al., 2009). In the QM:Caspr4 X-ray structure, the N-terminal peptide residue is disordered. Here, its position was adapted from the WT:Sdc1 complex, then adjusted through energy minimization and restrained MD. The ten other Caspr4 complexes were based on this model. All peptides had a neutral, N-terminal, acetyl capping group.

### 2.3. Molecular dynamics simulations and use of restraints

For each complex, we ran an MD simulation with explicit solvent. Starting structures were taken from one of the available crystal complexes. Mutations appropriate for each complex were introduced using the Scwrl4 program (Krivov et al., 2009). The structural model was energy minimized through 1,000 steps of conjugate gradient minimization. The resulting complex was immersed in a large box of water and waters overlapping the protein were eliminated. The solvated system was truncated to the shape of a truncated octahedral box using the Charmm graphical interface or GUI (Jo et al., 2008; Brooks et al., 2009). A few sodium or chloride ions were included to ensure overall electroneutrality. Protonation states of histidines were assigned to be neutral, based on visual inspection of hydrogen-bonding patterns in the 3D structure and calculations with the PropKa program (Bas et al., 2008; Olsson et al., 2011). MD was done at room temperature and pressure using a Nose-Hoover thermostat and barostat (Nose, 1984; Hoover, 1985). Long-range electrostatic interactions were treated with a Particle Mesh Ewald (PME) approach (Darden, 2001). The Amber ff99SB forcefield was used for the protein (Cornell et al., 1995) and the TIP3P model (Jorgensen et al., 1983) was used for water. Unless otherwise mentioned, simulations were run for 40–100 ns, depending on the complex, using the NAMD program (Phillips et al., 2005).

An important feature of the MD simulations is that they included a weak, non-invasive restraint energy term that maintained the N-terminal peptide residue close to the PDZ protein. The restraint energy was zero, except when the peptide moved beyond a certain threshold of separation, about 3 Å, at which point the restraint energy began to increase harmonically (flat-bottomed restraint) with a 3 kcal/mol/Å^2^ force constant. Indeed, in trial simulations, we found that this part of the peptide occasionally broke away from the protein, rebinding later. This can lead to large energy fluctuations, impossible to sample adequately in 100 ns. Rather, we make the hypothesis that the relative binding affinities of the different complexes can be estimated by scoring only the fully bound conformation. For 16 of the 51 complexes, even with the weak N-terminal restraint, the structure shifted away from the initial, native-like geometry, leading to a distorted conformation. Our single-trajectory PB/LIE free energy function is not expected to accurately score structures that present large differences in structure, for reasons discussed further below. Therefore, we applied additional, weak, flat-bottomed restraints to these complexes, which kept them within the native conformational basin. We expect that by scoring native-like conformations, we may underestimate slightly the binding affinity. The mean restraint energies were included in the bound state free energy estimate. They were less than 0.30 kcal/mol, except for one very weak binder (0.66 kcal/mol) and one other complex (0.39 kcal/mol).

### 2.4. Poisson-boltzmann and generalized born calculations

With the MD trajectories in hand, the electrostatic contribution Δ*G*_elec_ to the peptide binding free energy was obtained by either a PB or a GB method. For a given snapshot from the MD, explicit waters were discarded and the electrostatic free energy was computed from continuum electrostatics, treating the protein and ligand as a single, homogeneous dielectric medium and the solvent as another. The same calculation was performed for the separate peptide and protein (with structures taken from the same MD snapshot), and the electrostatic binding free energy was computed. Calculations were done for over 1,000 snapshots, 20 ps apart along each MD trajectory, and averaged. With PB, the electrostatic potential was calculated for each structure by solving the PB equation numerically, using a cubic grid and a finite-difference algorithm, implemented in Charmm (Im et al., 1998). The grid included 181 planes in each direction, with a 0.8 Å spacing between planes. The source charges were the atomic charges from the molecular mechanics force field (Amber ff99SB, see above). The potential on the outer grid boundary was approximated as the Debye-Hückel potential produced by these charges. For each structure, a second calculation was then performed using a smaller grid, with a 0.4 Å spacing, with the potential on the grid boundaries derived from the first calculation. The ionic strength was 100 mM (monovalent salt concentration). The solvent and solute dielectric constants were set to 80 and 8, respectively. GB calculations were done using a modified version of the Xplor program (Brünger, 1992; Moulinier et al., 2003) that implements the GB variant developed by Hawkins et al. (1995), which is very similar to the main variants used in the Amber software (Onufriev et al., 2002). It was optimally parameterized earlier for use with the Amber atomic charges (Lopes et al., 2007).

The statistical errors of the computed free energies were obtained by splitting each MD trajectory into batches of 5 ns each, giving *N* batches. Denoting *var*(Δ*G*) the variance of the *N* corresponding Δ*G* values, the uncertainty estimate for Δ*G* was then σ(Δ*G*) = $\sqrt{var\left(\Delta G\right)/\left(N-1\right)}$ and the uncertainty estimate for the relative binding free energy ΔΔ*G* was computed by adding the variances for the complex of interest and the reference complex WT:Sdc1. All the uncertainties were between 0.1 and 0.2 kcal/mol, suggesting the simulation lengths were sufficient.

For two complexes, WT:Sdc1 and QM:Caspr4, we also computed the PB contribution to the binding free energy using a three trajectory approach. Separate MD trajectories were performed for the complex and the separate partners and solute structures were extracted at regular intervals. The PB binding free energy was then computed by summing three contributions: (1) the free energy Δ*G*_bound_(ϵ_*W*_ = 80 → ϵ_*W*_ = ϵ_*P*_) to change the dielectric constant of the solvent from 80 to ϵ_*P*_; (2) the free energy Δ*G*(bound → unbound) to separate the partners, with the solvent dielectric constant set to ϵ_*P*_; (3) the free energy to restore the solvent dielectric constant to its usual value ϵ_*W*_ = 80 for the unbound partners. Contributions (1) and (3) were computed by solving the PB equation with Charmm. Contribution (2) was computed with Charmm by taking the Coulomb energy difference between a bound conformation (from the bound simulation) and an unbound conformation (from the separate PDZ and peptide simulations), dividing by ϵ_*P*_, and averaging over the MD conformations. The MD trajectories were of length 500 ns (complex and unbound protein) or 400 ps (unbound peptide). Conformations were taken every 40 ps. PB calculations were done as above.

### 2.5. Peptide preorganization free energy

**Figure 1:**
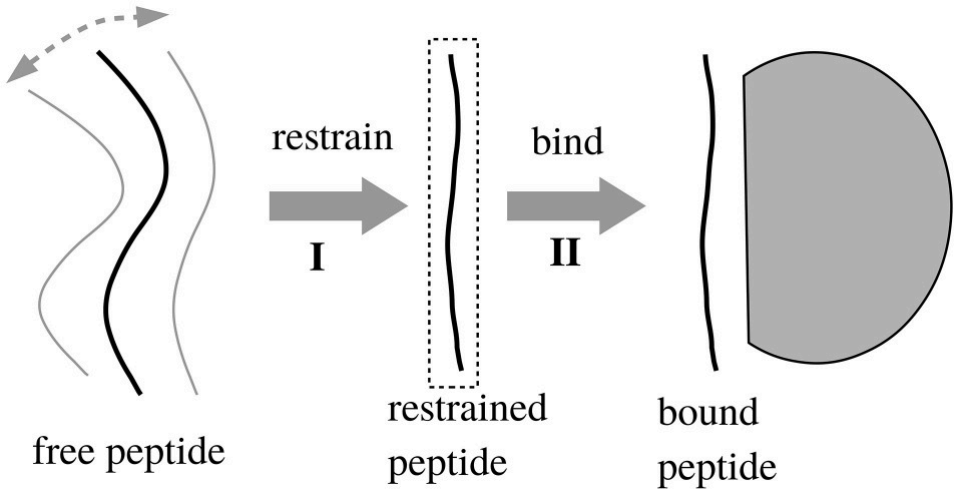
Two-step binding process. In step 1, we restrain the unbound peptide so that it occupies its bound conformation; in step 2 it binds. We assume that removing the restraints in the bound state does not affect the free energy.

We also considered a 2-trajectory model that explicitly takes into account the structural reorganization of the peptide upon binding. With this model, the binding reaction is divided into two steps, schematized in Figure 1: (I) first, the peptide is restricted to be in an extended conformation that will fit into the binding pocket; (II) second, the binding occurs. The free energy for the second step should be captured by the single trajectory of the bound state, since the unbound → bound structural changes are small by construction (small backbone adjustments and side chain reorientations). The free energy for the first step, Δ*G*_*I*_, was computed by considering a long MD simulation of the unbound peptide and deducing what fraction of time *f* it spent in the extended conformation. To determine the binding free energy difference between two peptides, *i* and *j*, to the same Tiam1 variant, let *f*_*i*_ and *f*_*j*_ be the extended fractions of the two unbound peptides. The contribution of step (I) to the binding free energy difference is given by

(2) $$\Delta \Delta G_{I}(i,j)=-kT\;\log f_{i}/f_{j}$$

To determine if the peptide was in the extended, bound conformation, we measured the backbone torsion angles ϕ, ψ of the five C-terminal peptide residues. If all the angles were within the β region of the Ramachandran plot (ψ ≥ 60° or ≤ −150° and ϕ ≤ −30°), the peptide was considered to be in the bound conformation.

### 2.6. Lazaridis-karplus non-polar free energy term

The PB/LIE and GB/LIE models (Equation 1) use the van der Waals and surface area terms to capture non-polar solvation effects. An alternate model replaces the SA term with a gaussian energy density proposed by Lazaridis and Karplus (1999):

(3) $$\begin{array}{l} \Delta G_{LK}=\sum_{i}G_{i}\\ \;G_{i}=G_{i}^{\text{ref}}-\sum_{j\neq i}\int_{V_{j}}g_{i}(r_{ij})dV\\ \;=G_{i}^{\text{ref}}-\sum_{j\neq i}g_{i}(r_{ij})V_{j} \end{array}$$

where the sum is over all solute atoms *i* and *V*_*j*_ is the volume of atom *j*. Each contribution reflects the transfer of atom *i* from a fully solvated state to its partially buried conformation within the solute. The free energy of the fully solvated atom *i* is given by an empirical reference value $G_{i}^{\text{ref}}$. The same atom within the solute is screened from solvent by the other solute atoms, which reduce the solvation free energy of atom *i*. This reduction is expressed as the integral of an energy density over the volume of the surrounding solute atoms. The energy density has a gaussian form:

(4) $$g_{i}(r_{ij})=\frac{G_{i}^{\text{free}}}{2\pi^{3/2}\lambda_{i}r_{ij}^{2}}e^{-{\left(r_{ij}-R_{i}\right)}^{2}/\lambda_{i}^{2}}$$

where *r*_*ij*_ is the interatom distance, *R*_*i*_ is the radius of atom *i*, and λ_*i*_ is a correlation length. The parameter $G_{i}^{\text{free}}$ is such that when *i* is fully buried, the total solvation free energy becomes zero. The overall free energy term has the form:

(5) $$\Delta G_{LK}=\sum_{i}G_{i}^{\text{ref}}-\sum_{i,j\neq i}g_{i}(r_{ij})V_{j}$$

In this work, the LK term Δ*G*_*LK*_ used parameters optimized elsewhere (Michael et al., 2017) and was multiplied by an adjustable weight γ.

### 2.7. Alchemical free energy simulations

The alchemical free energy simulation approach was used to calculate the binding free energy differences between several pairs of peptides that differed at a single position. To describe the method, we assume one peptide is the wildtype Sdc1 peptide while the other is a point mutant. For this pair, we considered a “hybrid” peptide, which has two side chains at the mutated position, one of each type. The corresponding energy function *U* depends on a coupling coordinate λ that scales selected electrostatic and van der Waals energy terms (Simonson, 2001). When λ = 0 (respectively, 1), the mutant (respectively, the wildtype) side chain was decoupled from its surroundings, retaining only its covalent interactions with its own backbone but not the protein or solvent. For intermediate λ values, both side chains were present, with intermediate weights. The van der Waals interactions of the side chain had a “soft-core” functional form, ensuring a very gradual insertion/deletion (Zacharias et al., 1994). Calculations were done with NAMD, using the “alch” facility (Phillips et al., 2005; Liu et al., 2012), with the parameter “alchElecLambdaStart” set to 0.4. 2 ns MD simulations (referred to as “windows”) were done for λ = 0, 0.1, 0.2, ⋯ , 0.9, 1. This set of 11 simulations (or windows) is referred to as a “run.” Each run was initiated from the endpoints of a previous run; typically, each λ window of run *i* was started from the endpoint of a nearby window of run *i* − 1: either the same or an “adjacent” λ value (λ±0.1). The first run for each system was initiated from MD simulations performed separately for the Sdc1 and mutant complexes. We usually ran 10–12 runs, totalling about 250 ns per system. The same procedure was applied to the unbound, solvated peptide. For each system, the free energy difference Δ*G*(λ → λ′) between two successive values of the coupling constant was computed using Bennett's Acceptance Ratio method (BAR) (Lu and Woolf, 2007), implemented through a perl script. Taking the difference between the free energy changes in the complex and the unbound peptide gave the binding free energy difference between the Sdc1 and the mutant peptide.

### 2.8. Experimental binding measurements

#### 2.8.1. Protein expression and purification

Wild-type Tiam1 PDZ protein expression was achieved in BL21(DE3) (Invitrogen) *Escherichia coli* cells. Typically, *E. coli* cells were grown at 37°C in Luria-Bertani (LB) medium supplemented with ampicillin (100 μg/mL) under vigorous agitation until an A600 of 0.6–0.8 was reached. Cultures were subsequently cooled to 18°C and protein expression was induced by the addition of isopropyl 1-thio-β-d-galactopyranoside (IPTG) to 1 mM final concentration. Induced cells were incubated for an additional 16–18 h at 18°C and harvested by centrifugation. The Tiam1 PDZ domain was purified by nickel-chelate (GE-Healthcare) and size-exclusion chromatography (Shepherd et al., 2010). The N-terminal His6 affinity tag was removed by incubation with recombinant tobacco etch virus (rTEV) protease for 36 h at 4°C. Undigested protein, cleaved His6 tag and His-tagged rTEV were separated from Tiam1 PDZ domain by nickel-chelate chromatography. The final yield was 20 mg of PDZ protein (>98% pure as judged by SDS-PAGE) from 1 L of culture. Samples were used immediately or lyophilized and stored at 80°C.

#### 2.8.2. Synthetic peptides for fluorescence anisotropy-based binding assays

All peptides were chemically synthesized by GenScript Inc. (Piscataway, NJ) and judged to be >95% pure based on analytical HPLC and mass spectrometry. Peptides were dansylated at their N-terminus. Peptide concentrations were determined by absorbance measurements (A280) using their predicted extinction coefficient. The peptides used in this study were Syndecan1 (Sdc1) point mutants and double mutants: Sdc1 (residues 303–310 of the full Syndecan protein: TKQEEFYA_*COOH*_), Sdc1-A0M (residues 303–310: TKQEEFYM_*COOH*_), Sdc1-F2R (residues 303–310: TKQEERYA_*COOH*_), Sdc1-F2R/A0M (residues 303–310: TKQEERYM_*COOH*_), Sdc1-F2A (residues 303–310: TKQEEAYA_*COOH*_), Sdc1-F2N (residues 303-310: TKQEENYA_*COOH*_), Sdc1-F2I (residues 303–310: TKQEEIYA_*COOH*_), Sdc1-E4L (residues 303–310: TKQLEFYA_*COOH*_), Sdc1-E3D/Y1T (residues 303–310: TKQEDFTA_*COOH*_), and Sdc1-E3T/Y1K (residues 303–310: TKQETFKA_*COOH*_). Notice that here and below, for the Sdc1 and other peptides, we number residue positions counting backwards from the peptide C-terminus (position 0). Thus, Sdc1-F2R is mutated at the third position from the C-terminus, which is usually called “position −2” (Shepherd and Fuentes, 2011). We drop the negative sign to keep the notation simple.

#### 2.8.3. *In vitro* binding measurements and thermodynamic analysis

Fluorescence anisotropy was used to monitor the binding of Tiam1 PDZ domain proteins to dansylated peptides. Anisotropy measurements were carried out on a Fluorolog3 (Jobin Yvon, Horiba, NJ) spectrofluorimeter (γ_*ex*_ = 340 and γ_*em*_ = 550 nm). All data collection, fitting, and thermodynamic analyses were performed as previously described (Shepherd et al., 2011). Binding experiments were conducted in 1.3 mL of binding buffer (20 mM sodium phosphate, 50 mM NaCl and pH 6.8) containing peptide at a concentration of 5–10 μM. Measurements were made in a 2 mL quartz cuvette that was stirred and maintained at constant 25°C temperature. The slit widths for the control of excitation and emission intensity were adjusted to the signal-to-noise ratio and maximum intensity and set in the range of 6–9 nm. A ratio of 1:100 and 1:10 dilutions of the stock PDZ protein solution (~1 mM) were prepared in binding buffer. For each experiment, 20–30 individual titration steps were performed until the sample had little or no change in anisotropy. The change in fluorescence anisotropy was plotted against protein concentration and fit to a standard hyperbolic ligand-binding curve. Each titration was carried out in triplicate.

## 3. Results

### 3.1. Target data set

Tables 1, 2 list the 44 peptide and protein variants for which experimental binding data are available along with the corresponding binding free energy differences (ΔΔ*G*) relative to the wildtype:Sdc1 complex (WT:Sdc1), taken as a reference. Notice that positions within each peptide ligand are numbered backwards from the C-terminus (“position 0”). For two complexes, experimental measurements were not available and ΔΔ*G* was obtained in this work using rigorous alchemical free energy simulations (see below). Seventeen of the complexes involve Tiam1 mutations at one or more of four positions. These include the four Tiam1 point mutants L911M, K912E, L915F, L920V, two double mutants, and the quadruple mutant (QM) (Shepherd et al., 2011). The WT:Sdc1 K_*d*_ is 26.9 μM, for a binding free energy of −6.70 kcal/mol (Shepherd et al., 2011). The strongest binding in the dataset is for the mutant L920V:Caspr4 complex, K_*d*_ = 10.8 μM, for a relative binding free energy ΔΔ*G* of −0.54 kcal/mol (relative to WT:Sdc1). For six “non-binding” peptides from the combinatorial library, only a lower bound for the binding free energy could be given: ΔΔ*G* ≥ 1.32 kcal/mol. For the peptides Sdc1-F2R, -F2A, and -F2N, the same lower bound was determined. For three other peptides, very weak binding could be measured, ΔΔ*G* = 1.67, 1.59, and 1.56 kcal/mol for Sdc2, Sdc4, and Sdc1-A0M, respectively. The other relative binding free energies were taken from earlier work (Shepherd et al., 2011; Liu et al., 2013) and were between −0.54 and 1.33 kcal/mol.

**Table 1:** Tiam1-PDZ:peptide complexes and free energies used for model fitting.

**Complex**	**Exp.^a^**	**Comp**.	**Error**	**PB**	**VdW**	**SA**	**Rest.^b^**	**Corr.^c^**
**Sdc1^d^**	0.00	0.00 (0.1)	0.00	0.00	0.00	0.00	0.00	0.00
Sdc1.A0F	0.43	0.16 (0.1)	−0.27	0.19	−4.21	−47.95	0.00	0.00
Sdc1.E4K	0.81	0.98 (0.2)	0.17	2.62	−0.17	−12.94	0.28	0.00
Sdc1.E4L	0.56^e^	0.49 (0.2)	−0.07	1.95	−1.30	−8.08	0.00	0.00
Sdc1.E3D, Y1T	0.87^e^	0.41 (0.1)	−0.46	1.67	2.08	12.05	0.00	0.00
Sdc1.E3T, Y1K	1.33^e^	0.38 (0.1)	−0.95	1.49	3.49	15.64	0.00	0.00
Sdc1.F2I	0.80^e^	0.26 (0.1)	−0.54	0.38	−0.44	−42.27	0.00	0.00
Sdc1.A0mA	0.04^f^	0.62 (0.1)	0.58	2.06	2.46	−13.51	0.00	0.00
Sdc3	0.13	0.21 (0.1)	0.08	0.38	1.38	−23.06	0.00	0.00
Consensus	0.84	0.48 (0.1)	−0.36	2.72	3.35	67.97	0.00	0.00
YAAEKYWA	0.72	0.36 (0.1)	−0.36	2.10	4.73	64.29	0.00	0.00
YAAKAFRF	1.17	1.35 (0.1)	0.18	4.70	5.02	53.73	0.29	0.00
YAAYRYRA	1.32^g^	1.26 (0.1)	−0.06	4.07	1.66	−18.18	0.14	0.00
YAARKFAK	1.32^g^	1.30 (0.1)	−0.02	3.81	7.13	5.44	0.23	0.00
YAAKRTYV	1.32^g^	1.14 (0.1)	−0.18	3.72	8.01	49.20	0.25	0.00
YAAGRKHF	1.32^g^	1.52 (0.2)	0.20	3.49	2.11	14.15	0.66	0.00
YAALIHKF	1.32^g^	0.98 (0.1)	−0.34	2.70	1.16	−2.87	0.27	0.00
YAAQKHFH	1.32^g^	0.92 (0.2)	−0.40	2.59	−1.74	−34.81	0.17	0.00
QM:CADM1	0.87	1.43 (0.2)	0.56	5.74	4.77	24.95	0.00	0.00
L911M:Sdc1	0.15	0.32 (0.2)	0.17	0.62	−1.03	−45.29	0.00	0.00
K912E:Sdc1	0.97	0.58 (0.2)	−0.39	2.27	−0.99	−8.66	0.00	0.00
L911M,K912E:Sdc1	1.21	0.54 (0.1)	−0.67	1.71	−1.52	−35.42	0.00	0.00
L915F:Sdc1	0.65	0.05 (0.2)	−0.60	0.04	−0.98	−13.78	0.00	0.00
L920V:Sdc1	0.31	−0.07 (0.2)	−0.38	−1.10	0.17	−48.56	0.01	0.00
L915F,L920V:Sdc1	1.32	0.19 (0.2)	−1.13	−0.24	1.03	−27.04	0.12	0.00
QM:Sdc1	0.89	0.27 (0.1)	−0.62	−0.02	−0.83	−71.82	0.00	0.00
QM:Sdc1.A0F	0.22	0.20 (0.2)	−0.02	0.26	1.50	−26.96	0.00	0.00
**QM:Casp^d^**	−0.23	−0.23 (0.1)	0.00	3.35	1.86	10.98	0.00	−1.06
WT:Casp	−0.21	−0.37 (0.1)	−0.16	2.04	2.77	32.77	0.26	−1.06
WT:Caspr.F0A	0.52	−0.57 (0.1)	−1.09	2.90	3.61	77.81	0.00	−1.06
L911M:Casp	−0.39	0.00 (0.1)	0.39	2.81	3.49	24.68	0.39	−1.06
K912E:Casp	0.46	0.09 (0.1)	−0.37	2.70	−0.44	−50.13	0.28	−1.06
L911M,K912E:Casp	0.04	−0.01 (0.1)	−0.05	3.41	2.57	22.74	0.24	−1.06
L915F:Casp	0.48	−0.45 (0.1)	−0.93	2.33	0.78	−2.68	0.00	−1.06
L920V:Casp	−0.54	−0.51 (0.1)	0.03	2.29	−0.28	4.82	0.00	−1.06
L915F,L920V:Casp	0.62	−0.37 (0.1)	−0.99	2.49	−0.07	−17.92	0.00	−1.06
QM:Caspr.F0A	1.09	−0.22 (0.1)	−1.31	4.59	3.78	95.58	0.00	−1.06

*Experimental and computed (PB/LIE) binding free energies, their deviation, and the computed components (PB, van der Waals, SA terms). Energies in kcal/mol; SA in Å^2^. Protein is WT unless otherwise mentioned. Peptide positions are numbered backwards from the C-terminus (position 0)*.

a *From earlier work (Shepherd et al., 2011; Liu et al., 2013) unless otherwise mentioned*.

b *Restraint energy*.

c *Free energy correction (Equation 6)*.

d *The Sdc1 sequence is TKQEEFYA. The Caspr4 sequence is ENQKEYFF*.

e *This work*.

f *From alchemical FEP simulations, this work*.

g *Lower bound*.

**Table 2:** Tiam1-PDZ:peptide complexes and free energies not used for model fitting.

**Complex**	**Exp.^a^**	**Comp**.	**Error**	**PB**	**VdW**	**SA**	**Rest.^b^**	**Corr.^c^**
Sdc1.A0M	1.56^d^	−0.05	−1.61	−0.51	−3.27	−36.08	0.00	0.00
Sdc1.A0V	1.90^e^	0.80	−1.10	1.30	−2.40	−81.36	0.20	0.00
Sdc2	1.67	0.37	−1.30	2.10	4.32	61.59	0.00	0.00
Sdc4	1.59	0.50	−1.09	1.69	4.46	59.45	0.23	0.00
QM:Neur	0.32	0.20	−0.12	0.70	3.98	12.96	0.00	0.00
L911M,K912E:Neur	1.25	0.29	−0.96	0.40	1.36	−39.92	0.00	0.00
L915F,L920V:Neur	1.08	0.17	−0.91	0.25	2.90	−11.94	0.00	0.00
Sdc1.A0Q	NA	0.18	NA	1.06	−2.72	7.76	0.00	0.00
Sdc1.F2C	NA	0.44	NA	1.15	0.81	−33.84	0.00	0.00
Sdc1.F2M	NA	0.01	NA	0.06	0.87	5.57	0.00	0.00
Sdc1.F2T	NA	0.17	NA	−0.05	−0.13	−45.20	0.00	0.00
Sdc1.F2V	NA	0.06	NA	−0.46	−1.09	−48.83	0.00	0.00
Sdc1.F2Y	NA	−0.04	NA	−0.57	−1.63	−33.98	0.00	0.00
Casp.F0mA	NA	0.09	NA	4.55	2.25	8.80	0.00	−1.06

*Free energies and components as in Table 1. Protein is WT unless otherwise mentioned. Energies in kcal/mol; SA in Å^2^*.

a *From earlier work (Shepherd et al., 2011; Liu et al., 2013) unless otherwise mentioned*.

b *Restraint energy*.

c *Free energy correction (Equation 6)*.

d *Measured in this work*.

e *From alchemical FEP simulations*.

**Figure 2:**
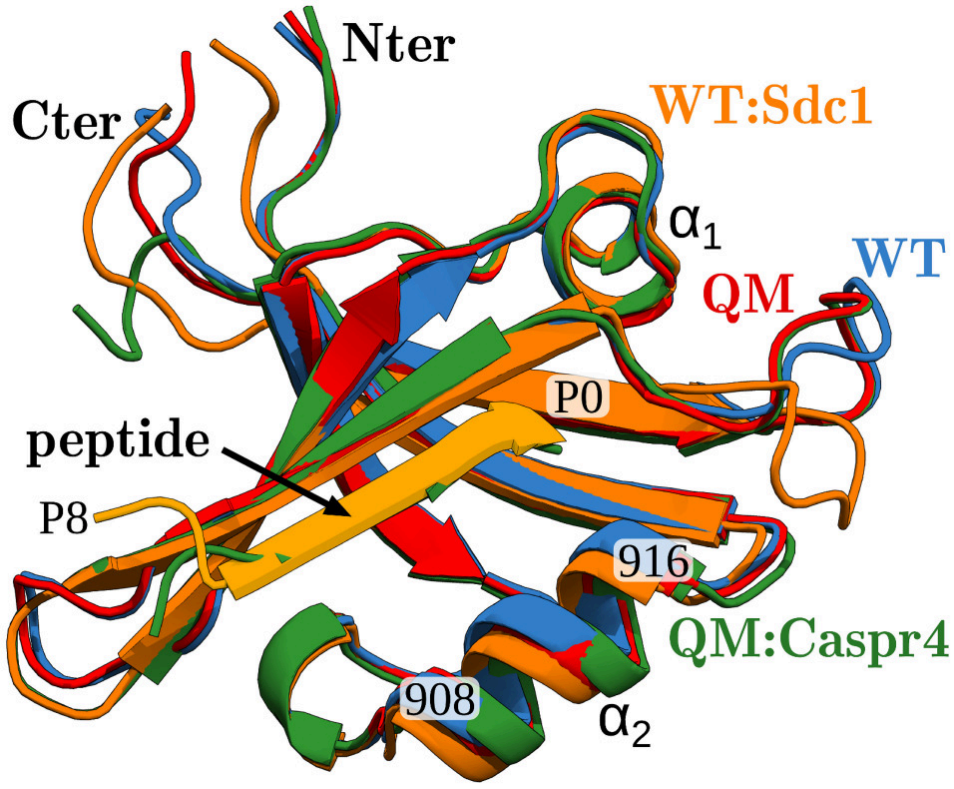
X-ray structures of the Tiam1 PDZ domain, ribbon view of backbone. Blue, apo-WT; orange, WT:Sdc1 (with peptide in lighter orange); red, apo-QM; green, QM:Caspr4. The peptide ligand, two helices, and certain residues are labeled. The peptide's last residue is labeled P0 (for position 0).

X-ray structures were available for the apo protein and the complexes WT:Sdc1, WT:consensus, QM:Caspr4, and QM:Neurexin1, shown in Figure 2. The corresponding peptide sequences are Sdc1: TKQEEFYA, consensus: SSRKEYYA, Caspr4: ENQKEYFF, Neurexin: NKDKEYYV. Backbone rms deviations between the four structures were in the range 0.5–1.5 Å (for the 19 residues within 5 Å of the peptide). Each of the other complexes was modeled using whichever of these four structures was deemed closest, by adopting the X-ray backbone and rebuilding the modified protein and/or peptide side chains with the Scwrl4 program (see Methods). Each complex was then immersed in a box of water and subjected to a molecular dynamics (MD) simulation. The trajectory length was between 40 and 100 ns (75 ns on average), depending on the rate of convergence of the free energy components, except for the WT:Sdc1 and QM:Caspr4 complexes, which were simulated for 500 ns. The backbone rms deviations between the 51 MD models were 0.6–2.1 Å. The deviations from the X-ray structures were 0.7–1.5 Å (for residues within 5 Å of the peptide).

Two groups of systems had a special treatment. (1) Four complexes with very weak binding (ΔΔ*G* > 1.5 kcal/mol) were excluded from the parameter fitting. Indeed, with such weak binding, we found that the peptide C-terminus tended to detach itself from the protein during MD unless restraints were applied. The single trajectory PB/LIE method assumes that conformational differences between complexes are small, and can be scored by a simple continuum dielectric model. This will not be the case if one complex is well-ordered and another undergoes large excursions of the peptide C-terminus. (2) Ten other complexes, involving the Caspr4 peptide, also exhibited a systematic structural difference compared to the others, related to the peptide N-terminus. Their structure was modeled based on the QM:Caspr4 crystal structure, where the peptide N-terminus is disordered. We positioned their N-terminal residues through model building and MD (see Methods), and this led to a distinct backbone conformation for these ten complexes. Therefore, we decided to score them relative to the QM:Caspr4 reference complex, instead of the WT:Sdc1 reference. This reduces the number of independent experimental observations by one. In practice, it is equivalent to correcting the computed ΔΔ*G* values for the ten Caspr4 complexes, by adding the experimental QM:Caspr4 ΔΔ*G* and subtracting the computed one:

(6) $$\begin{array}{l} \Delta \Delta G_{\text{comp}}(X)\prime =\Delta \Delta G_{\text{comp}}(X)+[\Delta \Delta G_{\text{expt}}(\text{QM }:\text{ Caspr4})\\ \;-\Delta \Delta G_{\text{comp}}(\text{QM }:\text{ Caspr4})] \end{array}$$

where X is a complex involving Caspr4 (or its F0A mutant).

In all, excluding the four very weak binders, there were 40 experimental free energies. Since WT:Sdc1 and QM:Caspr4 were taken as reference systems, we were left with 38 ΔΔ*G* values. Three complexes involving the Neurexin peptide were set aside for testing (Table 2). The other 35 values formed the experimental target data for fitting the free energy models (Table 1).

### 3.2. Model fitting

**Figure 3:**
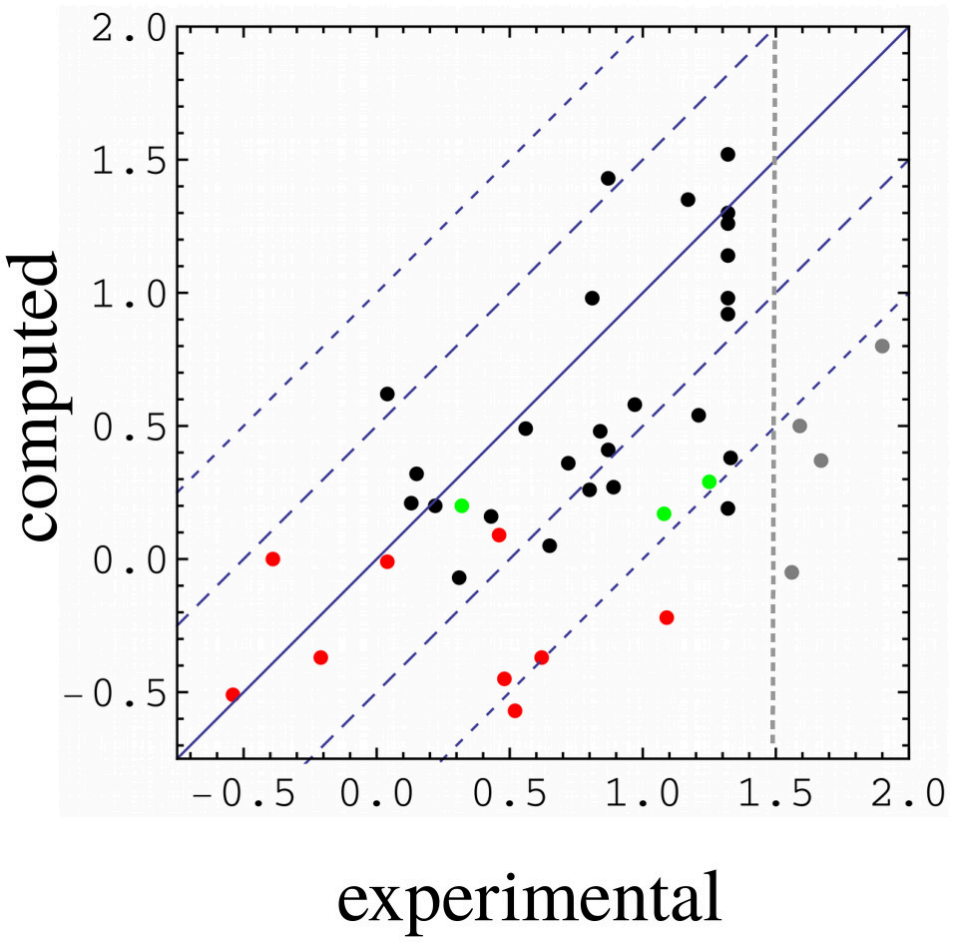
Computed vs. experimental relative binding free energies (kcal/mol). WT:Sdc1 is the reference system. Red, complexes involving Caspr4; green, complexes involving Neurexin; gray, complexes with very weak binding (not included in parameter fitting); black, all other complexes. The vertical line separates the weakest binders from the others. All but the weak binders (gray) and the three Neurexin complexes (green) were used for model fitting.

The free energy models included three adjustable parameters: the coefficients α, β, and γ (Equation 1). They were chosen to minimize the rms deviation between the 35 experimental and computed relative binding free energies in our target data set. With the PB electrostatic treatment, the optimal coefficients were α = 0.020 (van der Waals term), β = 0.25 (electrostatic term), and γ = −4 cal/mol/Å^2^ (surface term). The negative γ value means that surface burial upon binding is unfavorable. Similar (Tounge and Reynolds, 2003) or somewhat larger (Zhou et al., 2001) α values were used in some earlier models. The electrostatic coefficient β is comparable to those used in several earlier models (Tounge and Reynolds, 2003; Huang and Caflisch, 2004; Kolb et al., 2008; Singh and Warshel, 2010), if one takes into account the solute dielectric constant used here (ϵ_*P*_ = 8). The experimental and computed free energies are compared in Figure 3. For ten complexes involving Caspr4, the computed values included a correction term (in brackets on the right of Equation 6). The model parameters and error statistics are given in Table 3. The mean experimental and computed ΔΔ*G* values are 0.70 and 0.40 kcal/mol, respectively, so that the PB/LIE model has a small systematic error, overestimating the binding affinities by 0.30 kcal/mol on average. This tendency is visible for the Caspr4 complexes and for the four very weak binders (not included in the model fit) (Figure 3).

**Table 3:** Free energy model parameters and error statistics.

**vdW α**	**Elec β**	**SA, LK γ**	**Energy terms**	**Model name**	**rmsd**	**mue**	**R**	**Err_max_**	**〈 Err 〉**
NA			NA	Null	0.52	0.44	Zero	1.1	Zero
0.000	0.26	−2	vdW+PB+SA	PB/LIE	0.55	0.44	0.64	1.1	−0.30
0.020	0.25	−4	vdW+PB+SA	PB/LIE	0.55	0.43	0.64	1.2	−0.30
0.014	0.14	−5	vdW+GB+SA	GB/LIE	0.66	0.55	0.56	1.3	−0.43
0.130	0.20	0.05	vdW+GB+LK	GBLK	0.69	0.59	0.54	1.3	−0.48

*Energies in kcal/mol; SA in Å^2^. Err_max_ is the average of the three largest unsigned errors*.

**Figure 4:**
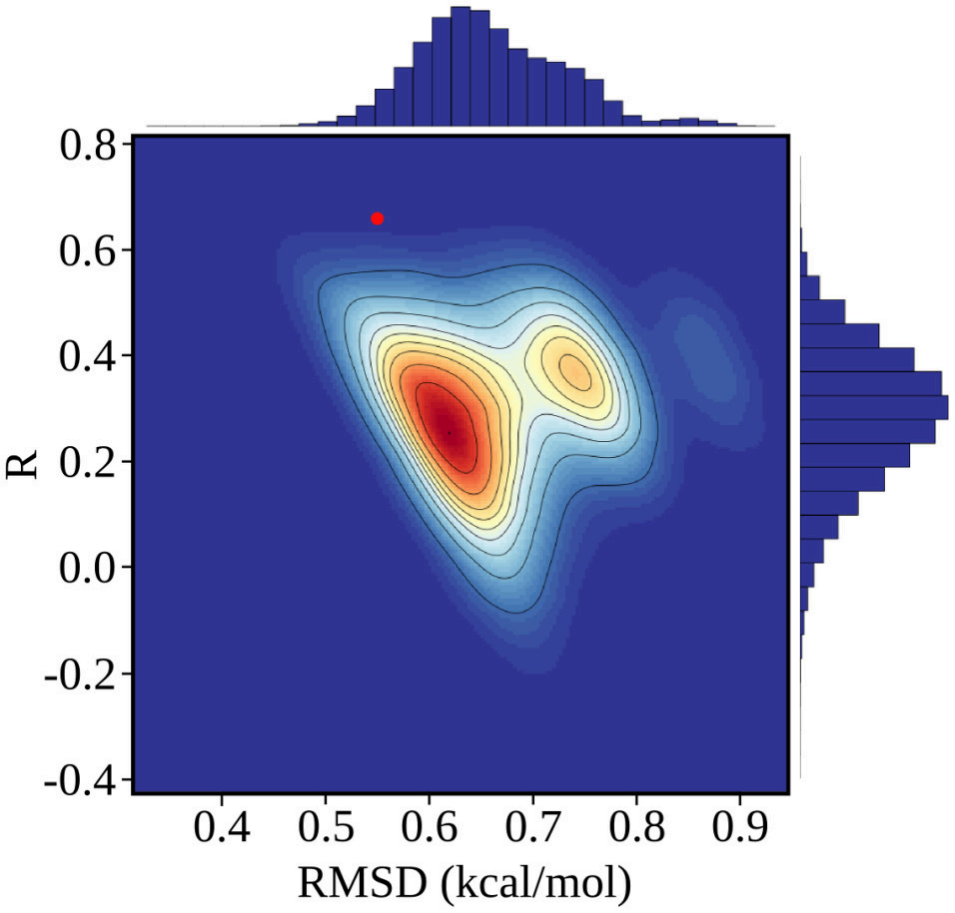
Error statistics for 100,000 random, Scrambled models obtained by fitting scambled experimental data. The number density of models in each region of the plot is color coded from red (high) to blue (low), and contour lines delimit regions that each contain 10% of the models. The PB/LIE model is shown as a red dot; only 63 scrambled models (0.06%) have better *R* and rmsd values. Histograms of *R* and rmsd values for the scrambled models are shown along the upper and right edges of the plot.

The rms and mean unsigned errors (mue) were 0.55 and 0.43 kcal/mol, respectively. The Pearson correlation between the experimental and computed values was *R* = 0.64. The three largest errors were 1.31, 1.13, and 1.09 kcal/mol and included two Caspr4 complexes. For the Caspr4 systems, the mue was 0.59 kcal/mol, slightly higher than the overall value. For comparison, we considered a Null model that assumes all the complexes have the same affinity. With this model, all the complexes have a ΔΔ*G* of 0.70 kcal/mol, which is the average of the 35 experimental values. The Null model gave an rms error of 0.52 kcal/mol and a mue of 0.44 kcal/mol, almost the same as the PB/LIE model, but with no correlation, by construction. The three largest errors with the Null model were 1.24, 1.09 and 0.91 kcal/mol and the mue for the Caspr4 systems was 0.56 kcal/mol. We also constructed 100,000 random, “Scrambled” models (Huang et al., 2006). These models were obtained by associating each experimental binding affinity with an arbitrary complex within our dataset—*not* the one for which it was measured—then adjusting α, β, and γ to minimize the rms deviation between the computed data and the scrambled experimental data. 99.94% of the Scrambled models gave larger errors and/or smaller correlations than the PB/LIE model (Figure 4).

Systematic cross-validation tests were done by partitioning the data into eight equal, random subsets, leaving out one subset and fitting to the remaining data. This was done for each subset, and led to error statistics for the omitted subset that were very similar to the overall errors: mue = 0.47 and rmsd = 0.55 kcal/mol, compared to 0.44 ± 0.03 and 0.56 ± 0.03 kcal/mol for the training sets, with free energy coefficients very similar to the overall fit: α = 0.02 ± 0.03, β = 0.25 ± 0.02, and γ = −4 ± 2 cal/mol/Å^2^. We also applied the model to three Neurexin complexes left out of the fit; the mue was 0.66 kcal/mol, compared to 0.44 kcal/mol for these complexes with the Null model.

With the GB electrostatic treatment, the error magnitudes were slightly larger than with PB/LIE, with a mue of 0.55 kcal/mol, an rms error of 0.66 kcal/mol, and a correlation of *R* = 0.56. The van der Waals free energy coefficient was larger: α = 0.14, the electrostatic coefficient was smaller: β = 0.14, and γ was similar (Table 3). The free energies predicted with PB/LIE and GB/LIE had a mutual correlation of *R* = 0.87 and a mutual rms deviation of 0.29 kcal/mol.

### 3.3. Alternate free energy models

#### 3.3.1. Alchemical free energy perturbation calculations

We applied a rigorous alchemical free energy perturbation method (FEP) to six complexes involving Sdc1 variants binding to WT, listed in Table 4. For four of them, A0M, A0F, F2I, and E4K, experimental binding constants were available. The other two were new: A0V and a variant where A0 had its H_α_ changed to a methyl. This gives an unnatural amino acid called α-amino isobutyric acid or α-methyl alanine, which we abbreviate Aib or mA. Such an amino acid might increase the peptide stability *in vivo* by providing protease resistance (Welch et al., 2007). For the four known variants, A0M, A0F, F2I, and E4K, agreement with experiment was excellent, with three ΔΔ*G* errors of 0.1–0.2 kcal/mol, comparable to the experimental uncertainty. For the fourth known variant, E4K, the error was larger, 0.9 kcal/mol. This variant involves a change of the peptide charge by +2, and the deviation from experiment might indicate a limitation of the force field employed. The E4K free energy includes a correction, described elsewhere (Lin et al., 2014; Simonson and Roux, 2016), for the use of a PME treatment of long-range electrostatic interactions and associated “tin foil” boundary conditions. The mue for the four variants is 0.4 kcal/mol.

**Table 4:** Alchemical free energy simulation (FEP) results.

**Peptide**	**Exp**.	**FEP**	**Error**
Sdc1.E4K	0.81	1.7	0.9
Sdc1.F2I	0.80	0.7	−0.1
Sdc1.A0F	0.43	0.5	0.1
Sdc1.A0M	1.56	1.8	0.2
Sdc1.A0V	NA	1.9	–
Sdc1.A0mA	NA	0.0	–

*WT binding free energy differences relative to Sdc1 in kcal/mol*.

The computed free energies are listed in Table 4. The good agreement with experiment indicates that the MD structures (which were also used for the PB/LIE modeling of the four known variants) are accurate. It also provides validation for FEP, which was then applied to two new variants. For Sdc1-A0V, the computed ΔΔ*G* of 1.9 kcal/mol indicates a very weak binding. This is consistent with the absence of Val at position 0 in the combinatorial library of Tiam1 binding peptides (Shepherd et al., 2011). For Sdc1-A0mA, FEP predicts a binding free energy equal to Sdc1, ΔΔ*G* = 0 kcal/mol, indicating that the Aib amino acid can be used for protease protection without loss of affinity. This last variant was included in the dataset used to fit the PB/LIE and GB/LIE models, above.

#### 3.3.2. Alternate non-polar treatments

**Figure 5:**
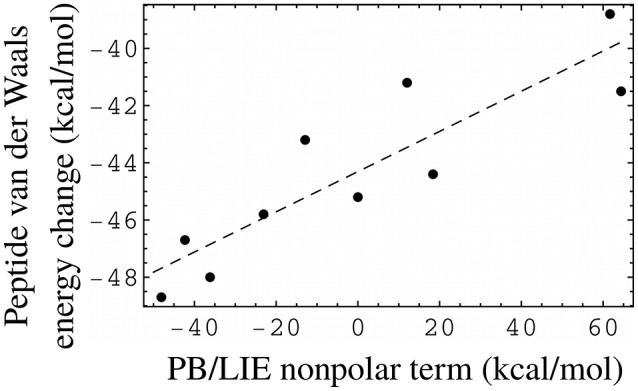
PB/LIE non-polar free energy term as a proxy for the peptide van der Waals energy change upon binding (including peptide–protein and peptide–solvent components). Each dot corresponds to one peptide, for which both bound and unbound MD simulations were available. The complexes all involve the WT protein. The dashed line is a linear fit (slope = 0.07), shown for clarity.

The PB/LIE model uses the protein–peptide van der Waals interaction energy (Δ*E*_vdW_ in Equation 1), without explicitly including solute–solvent or solvent–solvent interactions. While the small PB/LIE errors support this approach, we also directly compared the non-polar PB/LIE terms (van der Waals + SA) to the solute van der Waals energy, which has been used in some other LIE models. The comparison was possible for ten peptides, for which bound and unbound MD trajectories were both available. Figure 5 shows the peptide van der Waals energy change upon binding for the ten peptides, and compares it to the PB/LIE non-polar free energy term, αΔ*E*_vdW_ + γΔ*A*. The two quantities are well correlated.

An alternate PB/LIE free energy model that omitted the van der Waals contribution altogether was also obtained. It gave the same rms and mean unsigned errors as the 3-term model (Table 3). It used a negative coefficient γ = −2 cal/mol/Å^2^ for the SA term, meaning that surface burial upon binding was penalized. In the absence of a van der Waals term, the PB term was then the only term that favored binding.

We also considered an alternative to the surface area free energy term (Lazaridis and Karplus, 1999; Michael et al., 2017). We refer to it as the Lazaridis-Karplus or LK model (see section Methods). Combining the GB, van der Waals and LK terms, we obtained an rms error of 0.69 kcal/mol, a mue of 0.59 kcal/mol, and *R* = 0.54, close to GB/LIE (Table 3).

#### 3.3.3. Alternate sampling methods

For two complexes, WT:Sdc1 and QM:Caspr4, we performed much longer MD simulations and applied a 3-trajectory method to the PB free energy component (see section Methods). The unbound peptides were simulated for 400 ns, the two complexes for 500 ns, and the unbound proteins for 1,000 ns each. The binding free energy difference between the two variants was computed to be 1.04 kcal/mol, in excellent agreement with the value of 0.99 kcal/mol obtained with the single trajectory method using the 500 ns simulation. These values both exclude the empirical correction defined in Equation (6). The single trajectory value is just 0.16 kcal/mol higher than the one obtained from the 100 ns trajectories, which is within the MD uncertainty estimated earlier (Table 1). Solvent–solute and solvent–solvent van der Waals interactions were treated implicitly, as above. While they could in principle be calculated with three MD trajectories, the appropriate values for the free energy coefficients α, β, γ are not known.

We also considered a 2-trajectory model that explicitly takes into account the structural reorganization of the peptide (but not the protein) upon binding. With the single trajectory model, the unbound peptide conformations were taken from the bound MD simulation, where the peptide has an extended, β strand conformation. Structural changes in the unbound state were described implicitly, through the solute dielectric constant. This implicit description is expected to be accurate for solutes that undergo only small rearrangements upon binding (Swanson et al., 2004; Simonson, 2013). If the peptide is highly unstructured in the unbound state, a more sophisticated, model may be necessary (Figure 1), where the binding process is divided into two steps, with two distinct free energy contributions. The first, “preorganization” contribution, Δ*G*_*I*_, is deduced from the fraction of bound conformations found in MD simulations of the unbound peptide (Equation 2). The second contribution is computed from the PB/LIE or GB/LIE model as before.

To apply the 2-trajectory model, we simulated 12 unbound peptides: 11 of the 16 peptides that have known WT binding free energies, plus one “non-binding” peptide for which only a lower free energy bound is known (Sdc1-F2R). Each peptide was subjected to two simulations of 100 or 200 ns ns each. The ΔΔ*G*_*I*_ values ranged from 0.0 (Sdc1) to 1.2 kcal/mol for the 12 peptides (0.7 kcal/mol on average; Table 5). The positive values indicate that the peptide variants are all slightly less structured in solution than the reference Sdc1 peptide. The uncertainties, estimated by comparing the two MD runs, were between ±0.1 and ±0.7 kcal/mol (±0.4 kcal/mol on average) except for one peptide. For the YAAEKYWA peptide, despite 400 ns of MD simulation, the Δ*G*_*I*_ uncertainty was ±1.6 kcal/mol. The 12 peptides participate in 25 complexes in our dataset. We fitted the free energy coefficients α, β, γ to this smaller dataset, with or without the Δ*G*_*I*_ contribution. When the Δ*G*_*I*_ contribution was included, the rms error did not improve, but increased from 0.60 to 0.68 kcal/mol, while the correlation *R* decreased from 0.52 to 0.26 (Table 3).

**Table 5:** Relative peptide preorganization free energies.

**Peptide**	**% Folded^b^**	**ΔΔ G_I_**
^a^Sdc1	5.8/3.8	0.0 (0.2)
A0F	3.4/0.3	0.9 (0.8)
A0N	3.6/0.6	0.7 (0.7)
A0M	4.0/0.8	0.6 (0.6)
E4K	5.0/3.2	0.1 (0.3)
F2I	1.1/1.1	0.9 (0.1)
E3D,T1T	4.6/0.6	0.6 (0.7)
Sdc2	2.0/0.8	0.8 (0.4)
Sdc3	0.7/0.6	1.2 (0.1)
^a^Caspr4	2.6/8.6	0.0 (0.5)
^a^Caspr4-F0A	5.7/1.6	0.3 (0.5)
^a^YAAEKYWA	8.4/0.1	1.0 (1.6)

*ΔG_I_ (kcal/mol) corresponds to step I in Figure 1. Here, we tabulate the relative values ΔΔG_I_, compared to Sdc1*.

a *These peptides were simulated for 2 × 200 ns, the others for 2 × 100 ns*.

b *The “folded” peptide has the extended conformation seen in the bound state*.

### 3.4. Analysis of selected structures and free energies

#### 3.4.1. Selected structures

The MD simulations were done with explicit solvent and provide structural models for all 51 complexes (Tables 1, 2). We briefly discuss a few of them. The Sdc1 peptide and three variants have the side chains Ala, Val, Met, and Phe, respectively, at position 0. The WT:Sdc1-A0V complex was not characterized experimentally, but alchemical free energy simulations (above) predicted a very weak binding, ΔΔ*G* = 1.9 kcal/mol, and Val0 is completely absent from the experimental combinatorial library (Shepherd et al., 2011). The Val side chain does not lead to obvious steric overlap or structural changes compared to the wildtype Ala0. However, eight PDZ complexes in the PDB that have Val at the peptide C-terminus all have a different orientation of their α_2_ helix, which may increase slightly the volume of the P0 binding pocket. The structure of the QM:Neurexin complex supports this idea, as the Neurexin peptide contains Val at position 0 and the α_2_ helix orientation is changed relative to that in WT Tiam1 PDZ (Liu et al., 2016). With the larger Met0 side chain, WT:Sdc1-A0M also binds very weakly, ΔΔ*G* = 1.56 kcal/mol. The MD model for this variant is supported by alchemical free energy simulations (above), which gave good agreement with experiment for ΔΔ*G*. In the MD simulations, the Met0 side chain interacts with Leu915 at the C-terminus of the α_2_ helix, which exhibits a partial unwinding of its last turn. The WT:Sdc1-A0F complex binds more strongly, ΔΔ*G* = 0.43 kcal/mol. The large Phe0 side chain also perturbs the α_2_ C-terminus, but this may be counterbalanced by a favorable stacking of Phe0 on the protein Phe860, which occurs 50% of the time during MD.

We studied several Sdc1 variants with mutations at position −2. The WT:Sdc1-F2I complex has a significantly weaker binding than wildtype Sdc1, with ΔΔ*G* = 0.80 kcal/mol. In the MD, Ile2 remains in the P_−2_ pocket with the same orientation as wildtype Phe. However, Ile has a greater mobility within the pocket, indicating a looser packing, and its interaction with Leu911 in the α_2_ helix induces some deformation of the helix. We also note that Ile has more possible rotamers than Phe, so that its loss of side chain entropy upon binding is probably greater than that of wildtype Phe. The Sdc1-F2R variant does not have a detectable affinity for WT, even though Arg is found at this position in the combinatorial library. During the MD, Arg2 interacts with the peptide Glu4 side chain, so that this side chain in turn does not interact as strongly with the protein. In particular, the Glu4–Arg871 salt bridge is present 46% of the time in WT:Sdc1 but only 3% of the time in WT:Sdc1-F2R. The WT:Sdc1-E4K complex has ΔΔ*G* = 0.81 kcal/mol, which reflects the loss of the Glu4–Arg871 and Glu4–Ser908 interactions, found in the WT:Sdc1 X-ray and MD structures. The E4K mutation also leads to repulsion with nearby Lys912, within the α_2_ helix. Finally, the Sdc1-E3T,Y1K double mutant binds WT very weakly, ΔΔ*G* = 1.33 kcal/mol. Thr3 rarely contacts the protein, whereas the wildtype side chain Glu3 interacts with Asn876 16% of the time. Lys1 leads to repulsion with Lys879, 9.0 Å away.

#### 3.4.2. Prediction of new peptide variants

**Figure 6:**
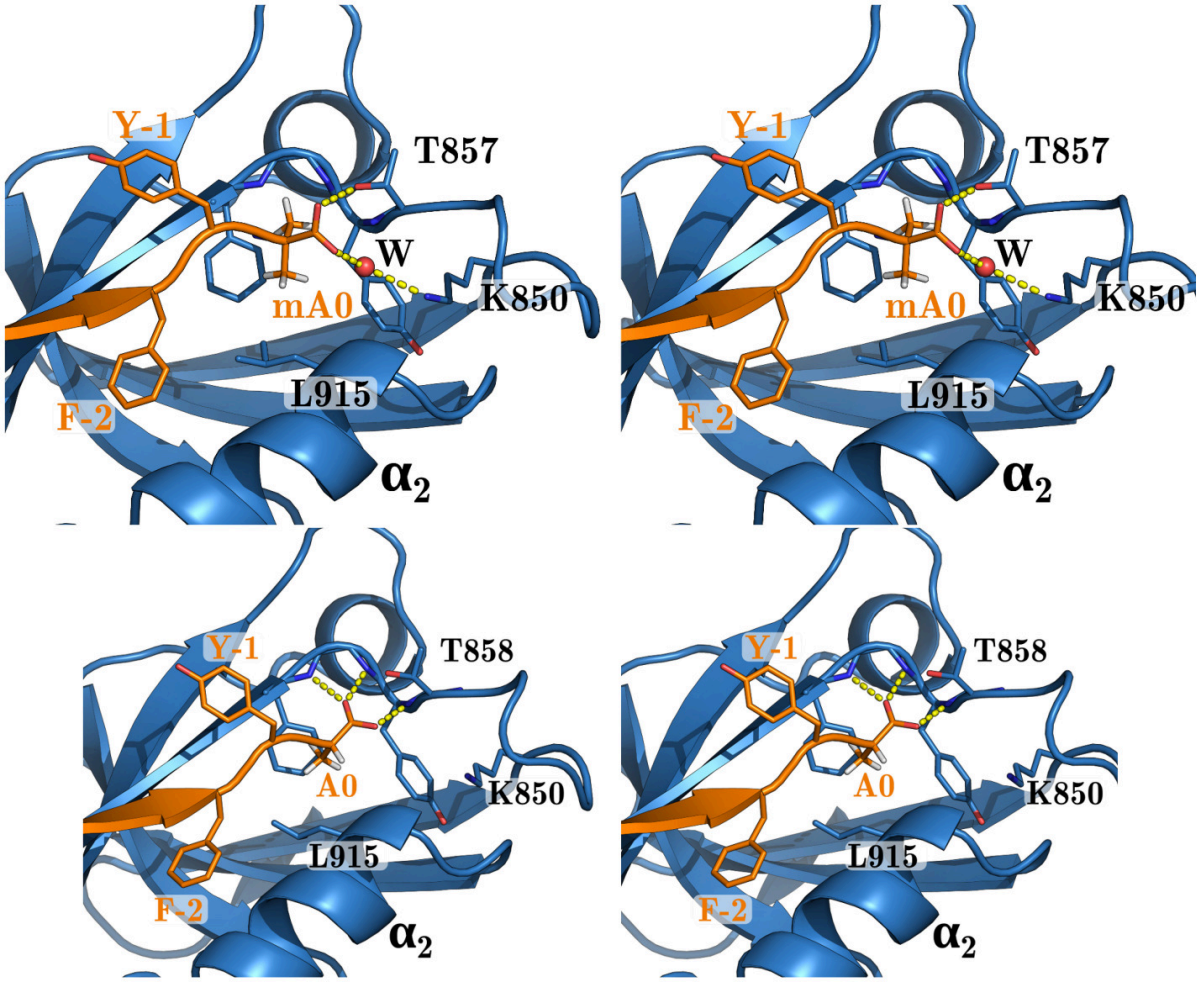
WT complex with Sdc1-A0mA (above) and Sdc1 (below). Closeup of the peptide C-terminus. Selected residues are labeled; peptide positions are numbered backwards from the C-terminus.

We explored Sdc1 variants binding to WT where Phe2 was mutated to Cys, Met, Thr, Val, and Tyr. For F2C, the predicted PB/LIE binding free energy was poorer than wildtype Sdc1 by 0.4 kcal/mol. For the other variants, the predicted binding free energies were the same as Sdc1 or slightly higher, 0.2 kcal/mol at most. We also predict that the unnatural amino acid Aib can replace Phe at the C-terminus of Caspr4 with a loss of affinity of just 0.1 kcal/mol compared to WT:Sdc1, or 0.3 kcal/mol compared to QM:Caspr4. This is very close to the binding free energy predicted by rigorous FEP calculations for Sdc1-A0mA. Such an amino acid could perhaps provide protease resistance and increase peptide lifetime *in vivo* (Welch et al., 2007). In the WT:Sdc1-A0mA complex, the peptide C-terminus has shifted slightly outwards, with a water molecule moving into the interface and forming hydrogen bonds to both the peptide C-terminus and the side chain of Lys850 (Figure 6). The shift was reproduced in several independent simulations.

## 4. Concluding discussion

The main goal of this study was to test and improve a class of simple free energy models for PDZ:peptide binding. Such models, if successful, can provide understanding and help design new peptide ligands. However, accurate calculation of protein:ligand binding free energies remains challenging, despite many efforts. Rigorous alchemical free energy perturbation approaches (or FEP) have no adjustable parameters but are costly, especially if one compares peptides that differ at multiple positions, like Sdc1 and Caspr4. Therefore, we focussed here on a class of semi-empirical models that are less expensive and can be applied to many complexes. They combined MD simulations of each complex in water (explicitly represented) and a free energy scoring function that uses continuum electrostatics and has a clear (albeit approximate) physical basis. Free energy models of this class have often been used, with both PB and GB solvents, with and without van der Waals and surface terms, with widely different choices for the solute dielectric constant, and sometimes with additional terms that describe hydrogen bonding or vibrational entropy. Several published variants are arguably unphysical, and there is a continuing need to clarify and test the validity and performance of this class of models.

Protein:peptide complexes have the advantage that well-established molecular mechanics force fields are available. They pose specific difficulties due to the large and complex binding interface and the flexibility of the unbound peptide ligand. We relied on 37 experimental binding affinities, measured for the wildtype and mutant Tiam1 PDZ domains, that included eight Sdc1 and two Caspr4 variants. The model had just three adjustable parameters. Several other variants were tried, with either GB or PB electrostatics and with either van der Waals and SA non-polar terms or a Lazaridis-Karplus non-polar term. We deliberately chose a strategy that used a single MD trajectory to model both the bound and unbound states, since a three-trajectory approach with separate bound and unbound simulations would have been several times more costly. With this approach, solvent van der Waals interactions were treated implicitly.

An important ingredient was the set of structural models constructed for the complexes that had no X-ray structure (all but four). A poor choice of side chain rotamers for a mutant position could lead to a bad free energy estimate. To ensure high-quality models, we tested model stability in rather long MD simulations (up to 100 ns). For four variants, rigorous alchemical FEP gave excellent agreement with experiment, validating the structures. A second important model ingredient was the use of restraints during the MD simulations. We applied very weak, flat-bottomed restraints to a few protein:peptide distances at the N-terminus of all the peptides, to suppress rare unbinding events that would not have been reliably scored with PB/LIE and 100 ns simulations. In addition, for ten complexes (out of the 37 used for model fitting), we used weak, flat-bottomed restraints to maintain the α_2_ helix in a native-like conformation, instead of a slightly distorted or bent one. It may seem disruptive to prevent the system from adapting its conformation in response to a mutation. However, the single trajectory free energy method can only compare two complexes if they have similar conformations; large conformational changes cannot be properly scored. By imposing a binding mode that is possibly suboptimal, we may underestimate the affinity of the ten complexes. This was partly or entirely compensated by including the restraint energy in the bound state free energy, and may have been further compensated by the empirical parameter optimization. We emphasize that the flat-bottomed restraints only acted when rare fluctuations away from the native-like structure occurred, and the mean restraint energies were very small.

Four especially weak (millimolar) binders (gray dots in Figure 3) could not be predicted reliably. In MD simulations, these complexes spent a significant amount of time in partly unbound conformations. Eliminating these conformations by applying restraints would be unrealistic, and would not accurately capture their conformational entropy or interaction energy, leading to an inaccurate score. Scoring the partly-bound conformations with PB/LIE would also be inaccurate, omitting much of the conformational entropy and oversimplifying the contributions from individual, partly-ordered water molecules located between the protein and peptide.

The PB/LIE model had three adjustable parameters and gave rms and mean unsigned errors of 0.55 and 0.43 kcal/mol, respectively, and a Pearson correlation of 0.64 for 35 experimental free energies. A Null model with one adjustable parameter gave similar mean errors but zero correlation. We can assume the deviations from experiment arise from a superposition of errors in the PB/LIE free energy model, statistical noise from the MD simulations, and random experimental errors. If we assume the mean experimental error is δ_exp_ = 0.2 kcal/mol and the MD simulations introduce a statistical error of δ_MD_ = 0.2 kcal/mol, and we denote σ_tot_ the rms deviation and δ_model_ the mean error in the free energy model, then we have $\sigma_{\text{tot}}^{2}$ = 0.55^2^ = $\delta_{\text{exp}}^{2}+\delta_{\text{MD}}^{2}+\delta_{\text{model}}^{2}$, which leads to a mean model error δ_model_ = 0.47 kcal/mol. This is about twice the experimental uncertainty and 1/4 of the experimental free energy range. Model variants that used a GB electrostatic term and a Lazaridis-Karplus non-polar term gave very similar results. A two-trajectory model that treated the peptide preorganization step explicitly did not give improved results, and had a significant uncertainty, despite 200–400 ns simulations of the unbound peptides. Estimates of the protein vibrational entropy based on the MD trajectories and the quasi-harmonic approximation were completely unreliable with the present (≤100 ns) trajectory lengths (data not shown). None of the model variants was able to score reliably the weakest, millimolar binders.

We expect our PB/LIE and GB/LIE models to be applicable to other complexes between the Tiam1 PDZ domain, its close homologs and a variety of peptide ligands. Model transferability to other, less homologous systems remains to be established. Similar LIE models have been applied to other systems, but with different values for the free energy coefficients, additional or fewer free energy terms, and/or different conformational sampling methods. Single-trajectory models with the same three free energy terms used similar values (Tounge and Reynolds, 2003) or larger values (Zhou et al., 2001) for all three coefficients. Similar values of the electrostatic coefficient β have been used in combination with larger van der Waals coefficients (α around 0.3) and no surface term (γ = 0) (Tounge and Reynolds, 2003; Huang and Caflisch, 2004; Kolb et al., 2008; Singh and Warshel, 2010). The small van der Waals coefficient used here multiplies the protein–peptide van der Waals energy. This free energy term along with the SA term can serve as a proxy (Figure 5) for the difference in van der Waals energies between the bound and unbound states, which is harder to compute as it requires at least two MD simulations per complex. The small α value used here can be understood as the result of cancellation between the bound and unbound contributions. Overall, while our free energy function differs from some earlier ones, its form is supported by the small errors obtained above, and it may be applicable to other protein–peptide complexes with a β-sheet interaction and possibly to other, less homologous systems.

We made PB/LIE predictions for seven complexes without experimental affinities and alchemical FEP predictions for two others. These did not reveal any candidates for strong peptide binding, but they suggest that an unnatural, Aib amino acid can be inserted at the C-terminus of both the Sdc1 and Casrp4 peptides with little or no loss of binding affinity. This could lead to an increased protease resistance and longer peptide lifetimes *in vivo*. The good model performance suggests that PB/LIE can be used in the future to search for new candidate peptides, and to filter or interpret experimental peptide libraries.

## Author contributions

TS and EF: Conceived study, interpreted data, and wrote paper. NP: performed simulations, interpreted data, and prepared figures. YS: performed experiments and interpreted data.

### Conflict of interest statement

The authors declare that the research was conducted in the absence of any commercial or financial relationships that could be construed as a potential conflict of interest.
